# A Case in Practice

**Published:** 1895-05

**Authors:** W. D. Miller

**Affiliations:** Berlin.


ARTICLE X.

### A CASE IN PRACTICE.

BY PROF. W. D. MILLER, M. D., D. D. S., BERLIN.

The question of alveolar abscess in connection with teeth containing living pulps, has gained very considerably in interest through the recent discussions on the etiology of pyorrhœa alveolaris.

When an abscess of this kind is now observed, it is put down as the result of a deposit of uric acid or urates in the pericementum, in consequence of a general gouty diathesis and is considered as evidence in support of the view that pyorrhœa alveolaris must have a similar origin.

The following case presents some points which seem to me well worth considering in this connection.

Herr X. presented himself with a left superior central incisor very slightly elongated and slightly looser than its perfectly healthy neighbor on the right side. The tooth was distinctly discolored. On the gums, about one-half an inch above the margin, was a marked, ridge-shaped hard prominence, from the center of which exuded on pressure a minute quantity of pus. This condition of things had existed for some years. The amount of pus evacuated was never considerable and sometimes it disappeared altogether for weeks.

Being sure of a putrid pulp, I trepanned the tooth from the lingual surface, but was very much surprised to run upon living tissue. The pain produced was not intense and was pronounced by the patient as "not feeling like the nerve." Nor did it at all produce upon me the impression.

The possibility has suggested itself to me that we have here a case where the chronic irritation at the apex of the root has produced absorption of the cementum and dentine, laying open the canal of the root and that granulations from the periosteum or bone marrow have grown into the canal as has been repeatedly observed in replanted or implanted teeth.

Nothing seems to indicate the presence of any deposit on the side of the root.

The patient is a young officer of most perfect health, in whose family there has never been a trace of gout.—*Proceedings of the American Dental Society of Europe.*

